# Clinical severity classes in COVID-19 pneumonia have distinct immunological profiles, facilitating risk stratification by machine learning

**DOI:** 10.3389/fimmu.2023.1192765

**Published:** 2023-09-05

**Authors:** Laura Wiffen, Leon Gerard D’Cruz, Thomas Brown, Tim W. Higenbottam, Jonathan A. Bernstein, Courtney Campbell, Joseph Moellman, Debajyoti Ghosh, Clive Richardson, Wynne Weston-Davies, Anoop J. Chauhan

**Affiliations:** ^1^Research and Innovation Department, Portsmouth Hospitals University National Health Service (NHS) Trust, Portsmouth, United Kingdom; ^2^School of Pharmacy & Biomedical Science, University of Portsmouth, Portsmouth, United Kingdom; ^3^AKARI Therapeutics PLC, London, United Kingdom; ^4^Department of Internal Medicine, Division of Rheumatology, Allergy and Immunology, University of Cincinnati College of Medicine, Cincinnati, OH, United States; ^5^Ohio State University Medical Centre, Department of Cardiovascular Medicine, Columbus, OH, United States; ^6^Department of Emergency Medicine, University of Cincinnati College of Medicine, Cincinnati, OH, United States

**Keywords:** COVID-19, biomarker, nomacopan, cytokine, machine learning, risk stratification, complement

## Abstract

**Objective:**

Clinical triage in coronavirus disease 2019 (COVID-19) places a heavy burden on senior clinicians during a pandemic situation. However, risk stratification based on serum biomarker bioprofiling could be implemented by a larger, nonspecialist workforce.

**Method:**

Measures of Complement Activation and inflammation in patientS with CoronAvirus DisEase 2019 (CASCADE) patients (*n* = 72), (clinicaltrials.gov: NCT04453527), classified as mild, moderate, or severe (by support needed to maintain SpO_2_ > 93%), and healthy controls (HC, *n* = 20), were bioprofiled using 76 immunological biomarkers and compared using ANOVA. Spearman correlation analysis on biomarker pairs was visualised *via* heatmaps. Linear Discriminant Analysis (LDA) models were generated to identify patients likely to deteriorate. An X-Gradient-boost (XGB) model trained on CASCADE data to triage patients as mild, moderate, and severe was retrospectively employed to classify COROnavirus Nomacopan Emergency Treatment for covid 19 infected patients with early signs of respiratory distress (CORONET) patients (*n* = 7) treated with nomacopan.

**Results:**

The LDA models distinctly discriminated between deteriorators, nondeteriorators, and HC, with IL-27, IP-10, MDC, ferritin, C5, and sC5b-9 among the key predictor variables during deterioration. C3a and C5 were elevated in all severity classes vs. HC (*p* < 0.05). sC5b-9 was elevated in the “moderate” and “severe” categories vs. HC (*p* < 0.001). Heatmap analysis shows a pairwise increase of negatively correlated pairs with IL-27. The XGB model indicated sC5b-9, IL-8, MCP1, and prothrombin F1 and F2 were key discriminators in nomacopan-treated patients (CORONET study).

**Conclusion:**

Distinct immunological fingerprints from serum biomarkers exist within different severity classes of COVID-19, and harnessing them using machine learning enabled the development of clinically useful triage and prognostic tools. Complement-mediated lung injury plays a key role in COVID-19 pneumonia, and preliminary results hint at the usefulness of a C5 inhibitor in COVID-19 recovery.

## Introduction

The novel severe acute respiratory syndrome coronavirus 2 (SARS-CoV-2) identified first in Wuhan, China, from human airway epithelial cells, was responsible for the coronavirus disease 2019 (COVID-19) (1). The clinical presentation of COVID-19 is highly variable (2); severe COVID-19 pneumonia presents with acute respiratory failure, septic shock, and multiorgan failure and may result in death (3).

Initial assessment and triage of patients by senior clinicians can often accelerate treatment *via* judicious planning and appropriate use of investigations; however, this places a huge burden on senior clinicians in a pandemic situation (4). However, triage strategies incorporating serum biomarker profiling, are implementable by nonspecialist and allied healthcare staff, thus reducing the bottleneck of waiting for senior clinician reviews.

During the first (March 2020–May 2020) and second (September 2020–April 21) waves of the pandemic, we risk-stratified COVID-19 patients by their clinical status and oxygenation requirements. A variety of cytokines, serum biomarkers, blood components, and complement proteins were evaluated in the CASCADE study (*n* = 72), initially to determine if there were “immunological fingerprints”, distinct to the clinical severities and thereafter to employ these in developing two predictive algorithms: the first, to identify patients likely to deteriorate clinically, and the second, a model to risk-stratify patients as “mild”, “moderate”, and “severe”. Since these models were trained on the levels of serum immunological proteins and not on viral proteins, the future applicability of the model may not be limited to COVID-19 infections caused by viral genotypic strains of the first and second waves.

The destructive role of complement in lung injury and COVID-19 pneumonia is well documented (5–7) and was also evident from this study (8). Complement is activated by SARS-CoV-2 *via* the lectin and classical pathways (9), while the SARS-CoV-2 spike protein (subunits 1 and 2) directly activates the alternative pathway (10).

Intuitively, therefore, complement inhibitors may have a role in influencing outcomes in the pandemic, and nomacopan, a complement C5 and leukotriene B4 (LTB4) inhibitor previously used in the treatment of paroxysmal nocturnal haemoglobinuria (PNH) (11) and bullous pemphigoid (12), was trialled in a small group of COVID-19 patients (CORONET study, *n* = 7), classified as “severe” using the model developed in the CASCADE study. The results from the CASCADE and CORONET studies are presented here, together with the analysis of biomarkers and the machine-learning algorithms developed as a consequence.

## Methods

### Study design, participant description, and approvals

**Figure 1:**
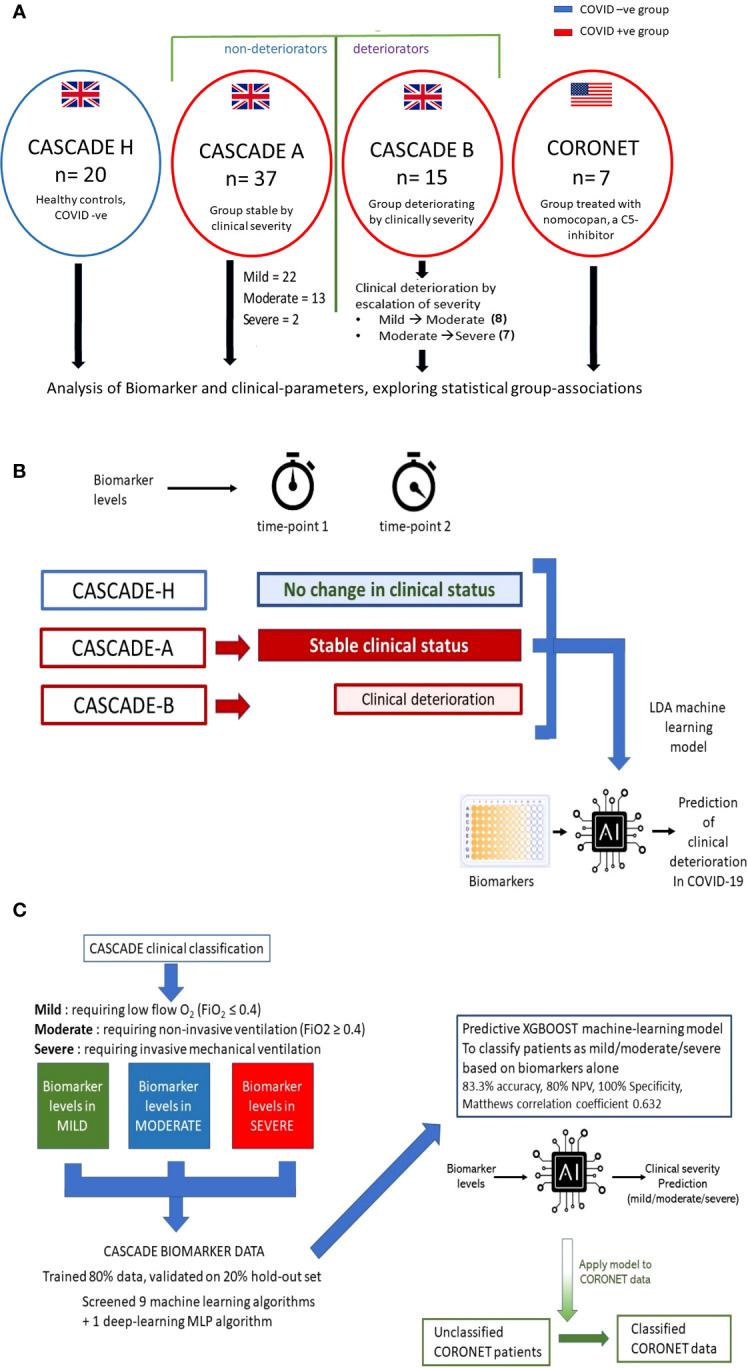
Outline of CASCADE and CORONET studies and description of predictive models generated. **(A)** Healthy controls (CASCADE H) were recruited from volunteers with stable, chronic medical conditions, including diabetes and hypertension, from nonclinical staff at Portsmouth Hospitals University NHS Trust (PHU). Participants in CASCADE A remained stable clinically throughout their hospital stay, whereas CASCADE B patients deteriorated clinically. In the event of deterioration with worsening respiratory failure, where patients transitioned to the next severity category (i.e., mild to moderate, moderate to severe) repeat blood samples were collected for biomarker analysis. Participants from the CORONET study were treated with nomacopan^®^. **(B)** The linear discriminant analysis (LDA) model was trained using an 80:20 split of the data, comprising CASCADE H participants, CASCADE A (patients who remained clinically stable throughout their hospital stay), and CASCADE B (patients who deteriorated at timepoint = 2). Admission biomarker data (timepoint 1) for CASCADE B patients were analysed initially together with the nondeteriorating group (CASCADE A) and healthy controls (CASCADE H) using LDA. Similarly, a second LDA classifier model was constructed using biomarker data from CASCADE B at point of clinical deterioration (time) compared to CASCADE A and CASCADE H participants. **(C)** Biomarker levels by clinical severity classes in the CASCADE study were used to train nine machine-learning and one deep-learning algorithm, the X-Gradient Boosting (XGBOOST) algorithm (trained and validated using an 80:20, train-test split) performed best (key performance metrics shown). The final XGBoost model was used to classify the CORONET patient bio-profile data retrospectively.

CASCADE was an observational cohort study that enrolled 52 COVID-19 quantitative polymerase chain reaction (qPCR)-positive patients admitted to the Portsmouth Hospitals University NHS Trust, and 20 qPCR-negative volunteers (CASCADE H), without comorbidities and those with stable, chronic medical conditions including diabetes and hypertension, composed of nonclinical hospital staff (**Figure 1A**).

The CORONET USA study enrolled five qPCR-positive COVID-19 patients from the University of Cincinnati Medical Centre (UCMC) and two qPCR-positive patients from the Ohio State University Medical Centre (OSUMC). The same inclusion and exclusion criteria were used for the CASCADE and CORONET studies (**Figure 1A**) and **Supplementary Table S1**.

The CASCADE trial details are summarised at “clinical trials.gov” [NCT04453527 (13)] and approved by the South-Central Berkshire Research Ethics Committee (REC reference: 20/SC/0228 22nd May 2020). The CORONET-study patients were treated with nomacopan in an open-label trial under a nonemergency “investigational new drug” (IND) application approved as an “expanded access application” by the FDA (13). Data and Safety Management Committees (DMSC) at each site provided safety oversight for this study.

All patients in both studies and control subjects in the CASCADE study provided written informed consent to obtain additional blood samples if their respiratory failure worsened, and CORONET patients also provided written informed consent to receive nomacopan.

### Nomacopan treatment

CORONET patients received a 45-mg subcutaneous dose of nomacopan on admission and two additional doses at 12-h intervals. After the initial three doses, nomacopan was administered at 45 mg every 24 h for a maximum of 12 days, unless they were discharged or passed away sooner. Patients were monitored for achieving normal 50% haemolytic complement (CH50) activity serum levels on day 3 or at discharge, similar to what has previously been reported for the C5 antibody, eculizumab (14, 15).

Patients received either a prophylactic beta-lactam or cephalosporin antibiotic (or an alternative agent if there was a penicillin or cephalosporin allergy) as terminal complement complex therapy has been linked to risk of meningococcal infection (16).

### Clinical severity of patients ranking in CASCADE

COVID-19 patients admitted in respiratory failure and recruited to the CASCADE study were ranked as mild, moderate, or severe respiratory failure according to the level of support required to maintain arterial oxygen saturation (SpO_2_) above 93%. Respiratory distress for patients was defined as mild if requiring low-flow oxygen (fraction of inspired oxygen; FiO_2_ ≤ 0.4), moderate if requiring FiO_2_ > 0.4, with or without noninvasive ventilation support (e.g., high-flow nasal oxygen or continuous positive airway pressure), and severe if requiring invasive mechanical ventilation.

For the CASCADE study, admitted patients who remained clinically stable entered the CASCADE A group (*n* = 37), and those who, on admission, deteriorated (moved between categories, from mild ➔ moderate or moderate ➔ severe or mild ➔ severe) entered the CASCADE B group (*n* = 15), summarised in **Figure 1A**. If CASCADE A patients demonstrated further clinical deterioration over 4 to 10 days after admission, they were reclassified to the CASCADE B group.

### Biological samples

COVID-19 qPCR nasal and throat swabs were obtained from all participants and processed according to standard published protocols (17) and as described in the **Online Data Supplement**. Blood samples obtained by venesection were handled using appropriate containment procedures (level 2). Serum and plasma samples were shipped on dry ice to external laboratories for biomarker analysis. Laboratory personnel were blinded to clinical information. Blood samples were obtained from 20 healthy controls (CASCADE H) and the 52 patients in the CASCADE A and CASCADE B groups for biomarkers.

### Statistics and machine-learning analysis

#### Heatmap analysis

A total of 76 biomarkers were analyzed in the CASCADE study. Spearman correlation coefficients between pairs of mean biomarker levels within each severity class of all patients and healthy controls in CASCADE were assembled into a 76 × 76 matrix. Further agglomerative hierarchical clustering and calculation of Euclidean distances between clusters were employed to build heatmaps (Seaborn API, ver 0.11.2). The colour map approaching hues of blue represents an inverse relationship between pairs of biomarkers, as one increases, the other variable decreases, moving in opposite directions while hues approaching red indicate positive (additive) correlation, where biomarker pairs change in their values together with the same sign.

#### Selection of machine-learning algorithms for analysis

To select the best machine-learning algorithm to address the analyses, nine machine-learning algorithms; K-nearest neighbour, ADA-Boost, Decision Trees, Random Forest, Extra Trees, Support Vector Classifier, X-Gradient Boost, Logistic Regression, and Linear Discriminant Analysis, and one multilayer perceptron (deep neural net) algorithm were screened using an iterative grid-search method in Python, using the training-dataset at the cross-validation step. The accuracy of prediction was employed as the metric. Hyperparameters for the chosen algorithm were then further optimised, further details are provided in the **Online Data Supplement**.

#### Linear discriminant analyses model for clinical deterioration in COVID-19

Two Linear Discriminant Analysis (LDA) models (model 1 for biomarkers collected only at timepoint 1 and model 2, biomarkers collected at timepoint 2) were generated in the CASCADE study, coded in Python 3.8, using Scikit-learn (ver.0.16.1). Timepoint 1 was at the time of recruitment and admission to hospital, and timepoint 2 was at the point of clinical deterioration. The model was trained and validated using an 80:20 train-test split. Hyperparameters were optimised and determined following extensive experimentation and testing of models while employing a grid-search method (18). Class imbalances were addressed using the Synthetic Minority Over-sampling Technique (SMOTE) method (19).

LDA machine-learning models explored if serum biomarker levels could identify and discriminate between patients who remained stable clinically and those who were likely to deteriorate, as described schematically in **Figure 1B**. Details are provided in the **Online Data Supplement**.

#### X-Gradient Boosting model for classification of mild, moderate, and severe in COVID-19

An X-Gradient Boosting (XGBoost) model was generated and trained on biomarker levels measured on CASCADE data from patients at different clinical severity levels *(* **Figure 1C***)*. The data were split into 80:20, train:test ratios. Nine machine-learning algorithms and one neural-net algorithm (multi-layer perceptron) were screened using the k-means stratified cross-validation (number of splits = 5) Hyperparameters were tuned using a grid-search method, and details of these are shown in the **Online Data Supplement**. Mean accuracy was used to evaluate the performance of the algorithms. The final validated model was used to retrospectively classify CORONET patients *(* **Figure 1C***)*.

#### Statistical analysis

All statistical analysis was carried out with SPSS version 25 (SPSS Version 25.0. Armonk, NY: IBM Corp), SciPy module (version 1.3) for Python (version 3.7.2) and R (version 3.60). Visualisation plots were created using Excel (Microsoft), Prism (GraphPad), or scripted using Python with Matplotlib (version 3.43) or Seaborn (version 0.11.2) software. Significance values in figures and tables are displayed using American Psychological Association (APA) styles (e.g., ns if *p* > 0.05; ^*^p ≤ 0.05; ^**^p ≤ 0.01; ^***^p ≤ 0.001).

## Results

### Demographics and baseline features of CASCADE and CORONET participants

**Figure 1A** summarises patient recruitment details. **Table 1** admission demographics compare patients in CASCADE A and CASCADE B COVID-19-positive groups with CASCADE H healthy controls. Patients in the CASCADE study were clinically triaged as mild, moderate, and severe. Patients under the mild category had an FiO_2_ ≤ 0.4, and those under the moderate category: FiO_2_ ≥ 0.4 and/or the use of noninvasive ventilation (NIV) or CPAP. Patients classified as severe were those who needed invasive/mechanical ventilation.

**Table 1:** Demographics and salient features of patients enrolled within the CASCADE (A and B) study.

	CASCADE H (*n* = 20)	CASCADE (A+B) COVID19 +ve (*n* = 52)	*p*-value	Mild	Moderate	Severe	*p*-value
Age	47.7 ± 13.5	64.4± 16.5	^***^	63.8 ± 16.2	65.5± 16.9	62.7 ± 16.4	ns
Gender (% male)	4 (20%)	34 (65%)	^***^	17 (57%)	21 (75%)	7 (78%)	ns
BMI	27.9 ± 5.1	32.4 ± 6.5	^**^	32.3 ± 6.6	31.9 ± 6.8	33.3 ± 6.6	ns
Smoking status							
Never (%)	6 (30%)	31 (57%)	^*^	20 (64.5%)	9 (45%)	2 (100%)	ns
Ex (%)	10 (50%)	22 (43%)	^*^	11 (35.5%)	11 (55%)	0	–
Current (%)	4 (20%)	0	–	0	0	0	–
Ethnicity							
Caucasian (%)	18 (90%)	50 (94)	ns	28 (90%)	20 (100%)	2 (100%)	ns
Asian (%)	1 (5%)	2 (4%)	ns	2 (7%)	0	0	ns
Afro-Caribbean (%)	1 (5%)	0	ns	0	0	0	ns
Other (%)	4 (20.0%)	0	ns	1 (3%)	0	0	ns
Comorbidities							
None	13 (65%)	20 (38.0%)		12 (39%)	7 (35%)	1 (50%)	–
1	6 (30%)	11 (20%)		7 (22%)	5 (25%)	0	–
≥2	1 (5%)	22 (42%)		12 (39%)	8 (40%)	1 (50%)	–
14-day outcome							
Discharged	–		–	24 (80%)	14 (50%)	1 (11%)	–
Inpatient	–		–	3 (10%)	8 (29%)	6 (67%)	^**^
RIP	–		–	3 (10%)	6 (21%)	2 (22%)	^**^

ns if p > 0.05; *p ≤ 0.05; **p ≤ 0.01; ***p ≤ 0.001.

CASCADE A were patients who remained stable within their clinical triage status before either recovery and discharge from the hospital or succumbing to death. CASCADE B had patients transitioning between categories of clinical severities (the deteriorating group), as summarised schematically in **Figure 1A**.

**Table 2** shows that age, gender, BMI, and length-of-stay were not statistically significant between the CASCADE A, CASCADE B, and CORONET groups by the Kruskal–Wallis test and, as such, were not considered confounding variables when comparing groups. Categorical variables such as sex, smoking status, comorbidities, and medication history were compared using crosstab and Chi-square analysis, *p* < 0.05 (95% confidence interval (CI)) was regarded as statistically significant. The average BMI of all participants across the three groups was in the range clinically classified as “obese” (BMI > 30). Participants in the nomacopan^®^ treatment group (CORONET) had a significantly higher proportion of diabetics (type 2 DM), compared to CASCADE A or CASCADE B. Participants in CASCADE A and CASCADE B had a significantly larger proportion of participants on long-term anticoagulation compared to the nomacopan^®^ treatment group. There was a higher proportion of participants on statins in the nomacopan^®^ group (*p* < 0.001) compared to the CASCADE A and CASCADE B groups.

**Table 2:** Baseline demographics, co-morbidities, and relevant medication history of participants in the study.

	(CASCADE A) nondeteriorating COVID-19 patients (*n* = 37)	(CASCADE B) deteriorating COVID-19 patients (*n* = 15)	(CORONET) patients on nomacopan (*n* = 6)	*p*-value
Sex (M:F)	25 (67.6%):12 (32.4%)	10 (66.7%):5 (33.3%)	6 (100%):0 (0%)	0.249
Age	65.25 ± 16.69	63.27 ± 17.32	50.0 ± 11.3	0.120
BMI	32.6 ± 7.7	31.9 ± 5.2	32.0 ± 3.8	0.856
Average length of stay (days)	11.0 ± 7.4	13.3± 5.9	6.0 ± 7.0	0.137
Smoking status				
Never smoked	20 (54.1%)	10 (66.7%)	4 (66.7%)	0.645
Ex-smoker	17 (45.9%)	5 (33.3%)	2 (33.3%)	
Current smoker	0 (0%)	0 (0%)	0 (0%)	
Comorbidities				
Diabetes	8 (21.6%)	3 (20.0%)	5 (83.3%)	0.005
Hypertension	12 (32.4%)	3 (20.0%)	4 (66.7%)	0.120
Heart disease	9 (24.3)	5 (33.3%)	0 (0%)	0.272
Chronic kidney disease	5 (13.5%)	3 (20.0%)	1 (16.7%)	0.840
Previous VTE	2 (5.4%)	2 (13.3%)	1 (16.7%)	0.496
Malignancy	3 (8.1%)	1 (6.7%)	1 (16.7%)	0.749
Immunosuppression	0 (0%)	1 (6.7%)	0 (0%)	0.233
Medication history				
Long-term anticoagulation	35 (94.6%)	13 (86.7%)	1 (16.7%)	<0.001
ACE inh/ARB	8 (21.6%)	4 (26.7%)	3 (50.0%)	0.337
Statins	12 (32.4%)	5 (33.3%)	3 (50%)	<0.001
Inhaled corticosteroids	3 (8.1%)	5 (33.3%)	1 (50%)	0.740

### CASCADE (mild, moderate, and severe) and CORONET patients are distinct, using ROX, NEWS2, and SOFA scores

The ROX index is a clinical assessment of the degree of a patient’s hypoxaemic respiratory failure and need for intubation. Normal ROX indices were calculated with FiO_2_ of 21%, respiratory rates between 12 and 18 breaths per minute, and SpO_2_ of 95%–98%. Patients in the severe category were intubated and received oxygen-enriched air, thus having a higher than atmospheric FiO_2_, this accounts for the higher mean value and the slightly elevated range of maxima and minima within the severe category compared to the moderate category.

**Figure 2:**
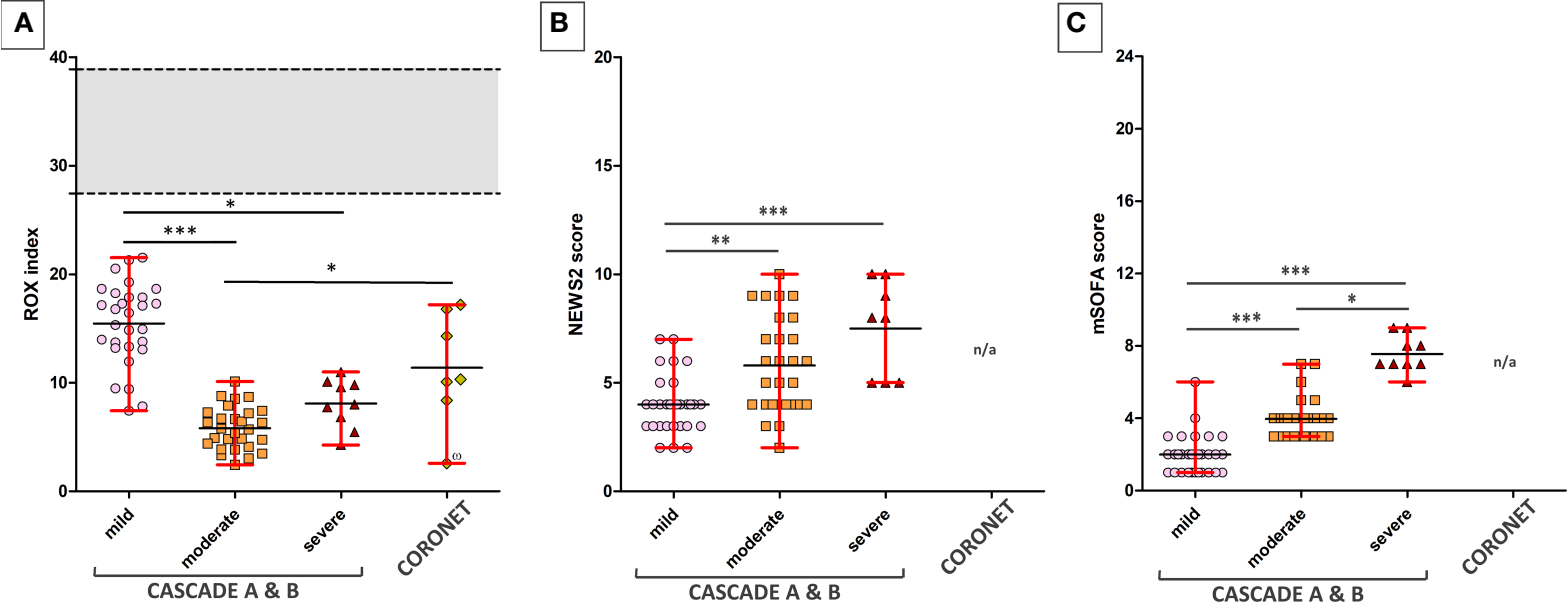
ROX indices of patients across various clinical severities in the CASCADE and CORONET studies. **(A)** The ROX index for normal, healthy individuals is shown as the grey area. The upper and lower limits shown in red bars represent the maximum and minimum data within that group. Black horizontal lines represent the average ROX index. CASCADE B comprised patients who deteriorated while in hospital, progressing either from mild to moderate or moderate to severe categories, while CASCADE A patients remained stable within that category. CORONET patients treated with nomacopan^®^ also had ROX indices below that of normal, healthy individuals. One patient in the CORONET study (data point marked with the number sign) had late commencement of treatment due to delays in reaching the treatment centre in Ohio, required invasive mechanical ventilation, and unfortunately died 13 days following admission due to COVID-19-related complications. **(B)** Mild, moderate, and severe patients in CASCADE grouped by NEWS2 scores **(C)** and grouped by mSOFA scores. Data for NEWS2 and mSOFA were not available (n/a) for CORONET patients. ns if p > 0.05; *p ≤ 0.05; **p ≤ 0.01; ***p ≤ 0.001.

When grouped by ROX indices, NEWS2, and mSOFA scores, participants in the clinical severity groups were significantly distinct from each other using Dunn’s multiple comparison tests (**Figure 2**). All seven patients in the severe category were mechanically ventilated.

### Analysis of markers of inflammation, coagulation, and complement

**Figure 3:**
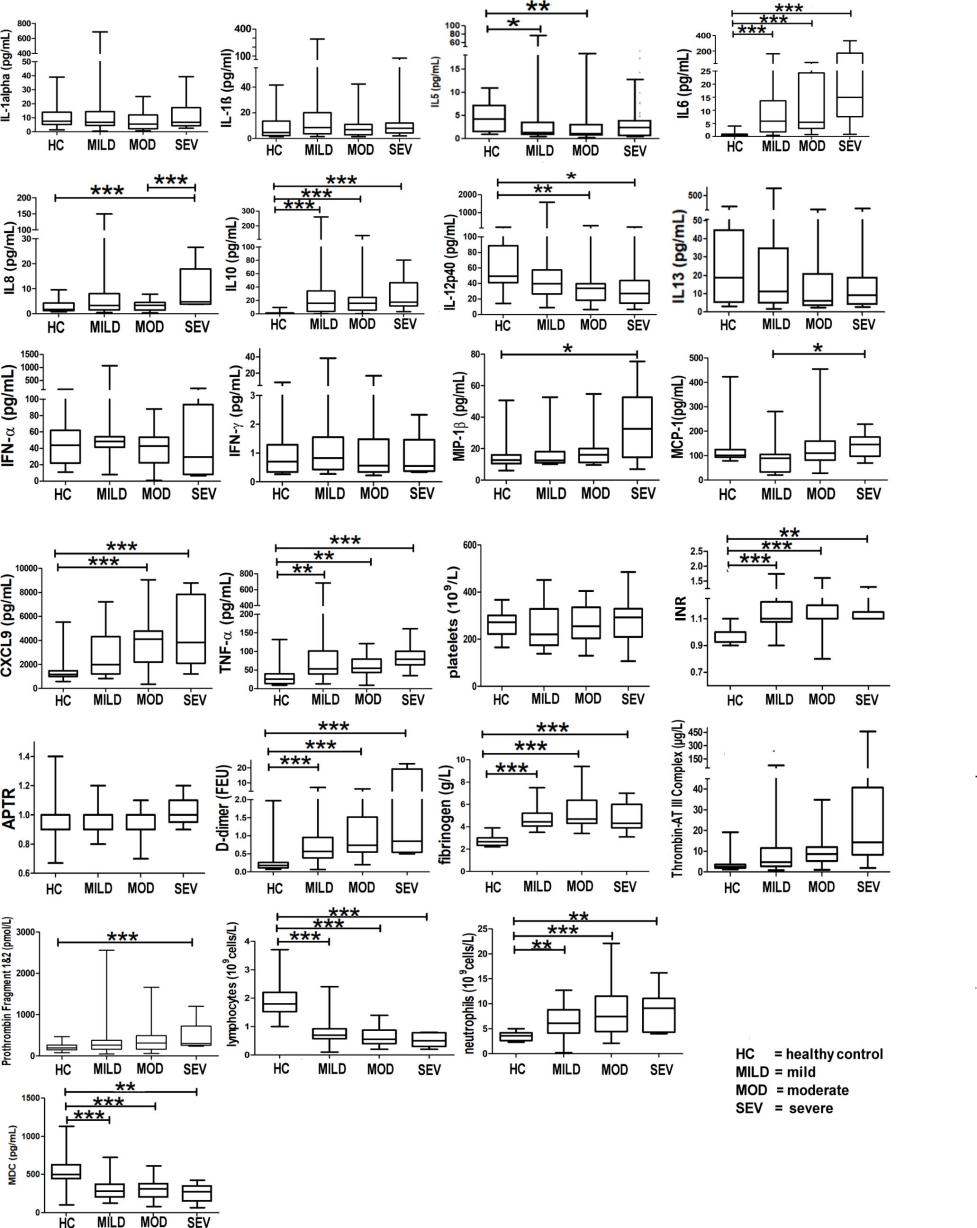
Markers of hyperinflammation and coagulation in healthy vs. COVID-19-infected patients. Mean values are shown as a horizontal line within the boxes; the whiskers indicate min and max ranges. (HC, healthy controls; MOD, moderate; MILD, mild; SEV, severe). ns if p > 0.05; *p ≤ 0.05; **p ≤ 0.01; ***p ≤ 0.001.

Markers of hyperinflammation, degree of lymphopaenia, neutrophil levels, TNF-α, INR levels, and proinflammatory cytokines (interleukin (IL)-6 and IL-8) were significantly increased in COVID-19 patients compared to healthy controls (*p* < 0.05, 95% CI) (**Figure 3**). In addition, D-dimer levels were significantly elevated in COVID-19 patients in the severe category (*p* < 0.05, 95% CI) above levels for normal-healthy individuals (normal reference ranges: <250 ng/ml).

IL-5 levels were significantly raised in the mild and moderate groups when compared to the healthy controls (**Figure 3**); IL-13 levels, however, did not show any difference between any of the groups (**Figure 3**).

**Figure 3** shows that patients infected with COVID-19 have deranged levels of IL-6, IL-8, IL-10, IL-12p40 (a component of IL-12 and IL-23 and a chemoattractant for macrophages to sites of inflammation), MIP-1b (responsible for macrophage chemotactic action), monocyte chemoattractant protein-1 (MCP-1, a cytokine related to thrombosis), chemokine (C-X-C motif) ligand 9 (CXCL9, a chemoattractant for activated T cells) and TNF-α (a proinflammatory cytokine, a target for immune biologics such as infliximab, adalimumab, or etanercept). INR levels in COVID-19 patients were significantly raised (normal healthy levels = 1.1). D-dimer levels were significantly raised (**Figure 3**), implying high circulating levels of fibrin degradation products, and concomitantly raised fibrinogen and prothrombin levels suggest a pro-coagulative state in COVID-19.

IL-27 levels appear low in healthy controls but are raised in all severity classes (**Figure 3**). Interestingly, the average IL-27 level appears to be lower in the severe class, compared to that in the mild and moderate classes, although the decrease does not appear to be significant (*p* > 0.05) (**Figure 3**). Interferon gamma-induced protein 10 (IP-10) appears to be raised significantly above the levels of healthy controls in all severity classes.

**Figure 4:**
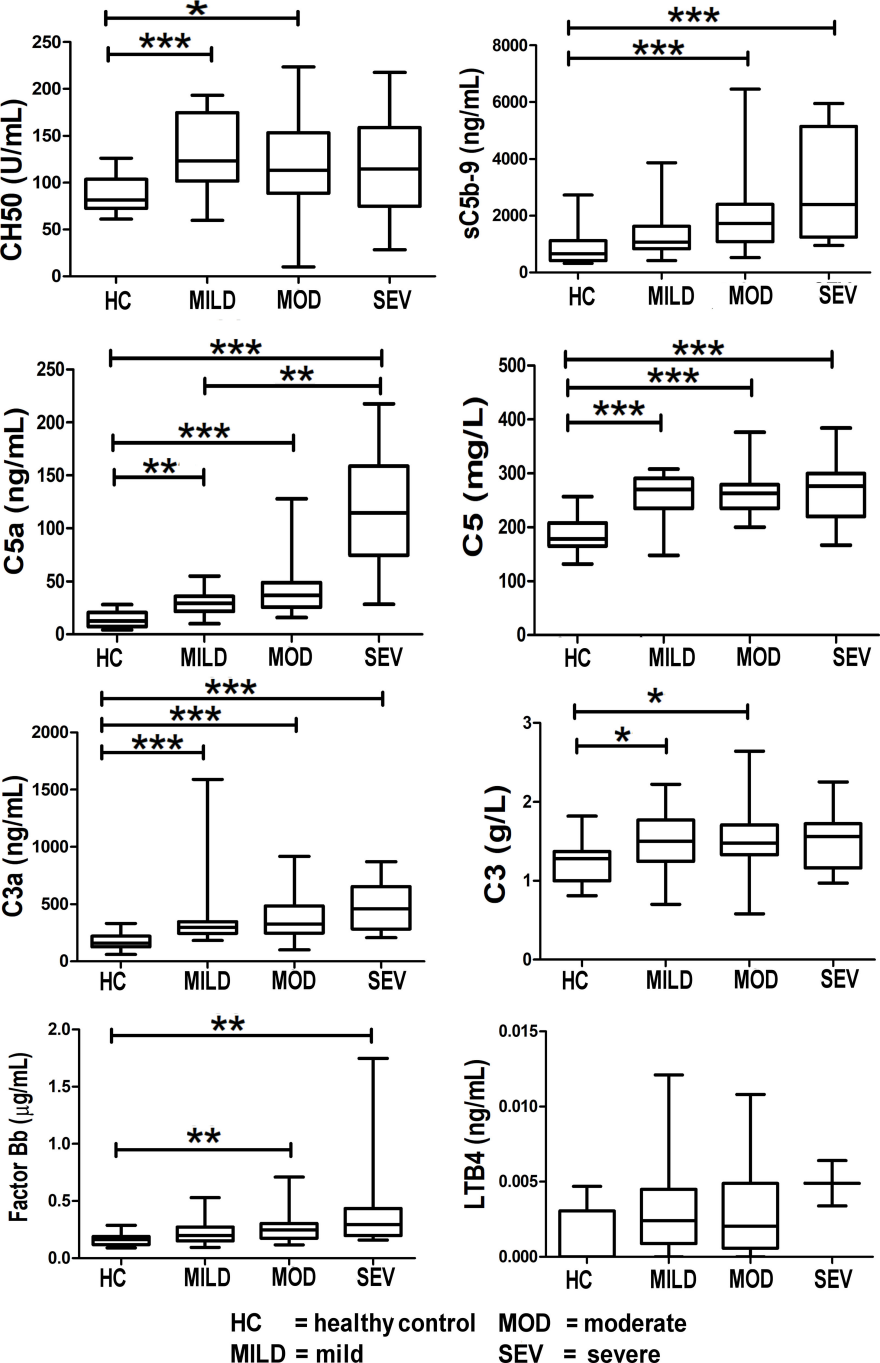
*Levels of complement components and LTB4 in healthy controls compared to COVID-19-infected patients.* Mean values are shown as a horizontal line within the boxes; whiskers indicate min and max ranges. Data were available for only two patients in the “severe” category for LTB4. (HC, healthy controls; MOD, moderate; MILD, mild; SEV, severe). ns if p > 0.05; *p ≤ 0.05; **p ≤ 0.01; ***p ≤ 0.001.

The complement components, C3a, C5a, and C5, were significantly elevated in all severities of COVID-19-infected patients (mild, moderate, and severe) with pneumonia compared to healthy controls (**Figure 4**). Of note, the C5a (*p* < 0.01) and sC5b-9 (*p* < 0.001) levels were significantly different between the moderate and severe categories compared to healthy controls.

Levels of the terminal complement complex, sC5b-9 (the membrane-attack complex (MAC)) were significantly raised in COVID-19 patients (**Figure 4**). Levels of C5a and C5 were raised in COVID-19 patients, thereby offering a rationale for C5 inhibitor therapy in COVID-19; this is reported in the results of the CORONET study. Levels of C3a (an anaphylatoxin and cleavage product in the formation of the C3 convertase) were significantly raised, strongly confirming complement cascade activation in COVID-19 (**Figure 4**). This is also reflected in the significant rise of factor Bb levels (**Figure 4**), the fragment together with C3b which forms the C3 convertase, a key step in the activation of the complement cascade.

Levels of leukotriene B4 (LTB4), a member of the eicosanoid family of lipid mediators, are also shown here (**Figure 4**). Although not part of the complement cascade, nomacopan^®^, the C5-inhibitor drug used in the CORONET study, binds tightly to LTB4. Limited samples were obtained for LTB4, and although these levels were higher in severe COVID-19 patients compared to stable COVID-19 patients and healthy controls, they were not statistically significant.

### Heatmap and correlation of biomarkers by disease severity

**Figure 5:**
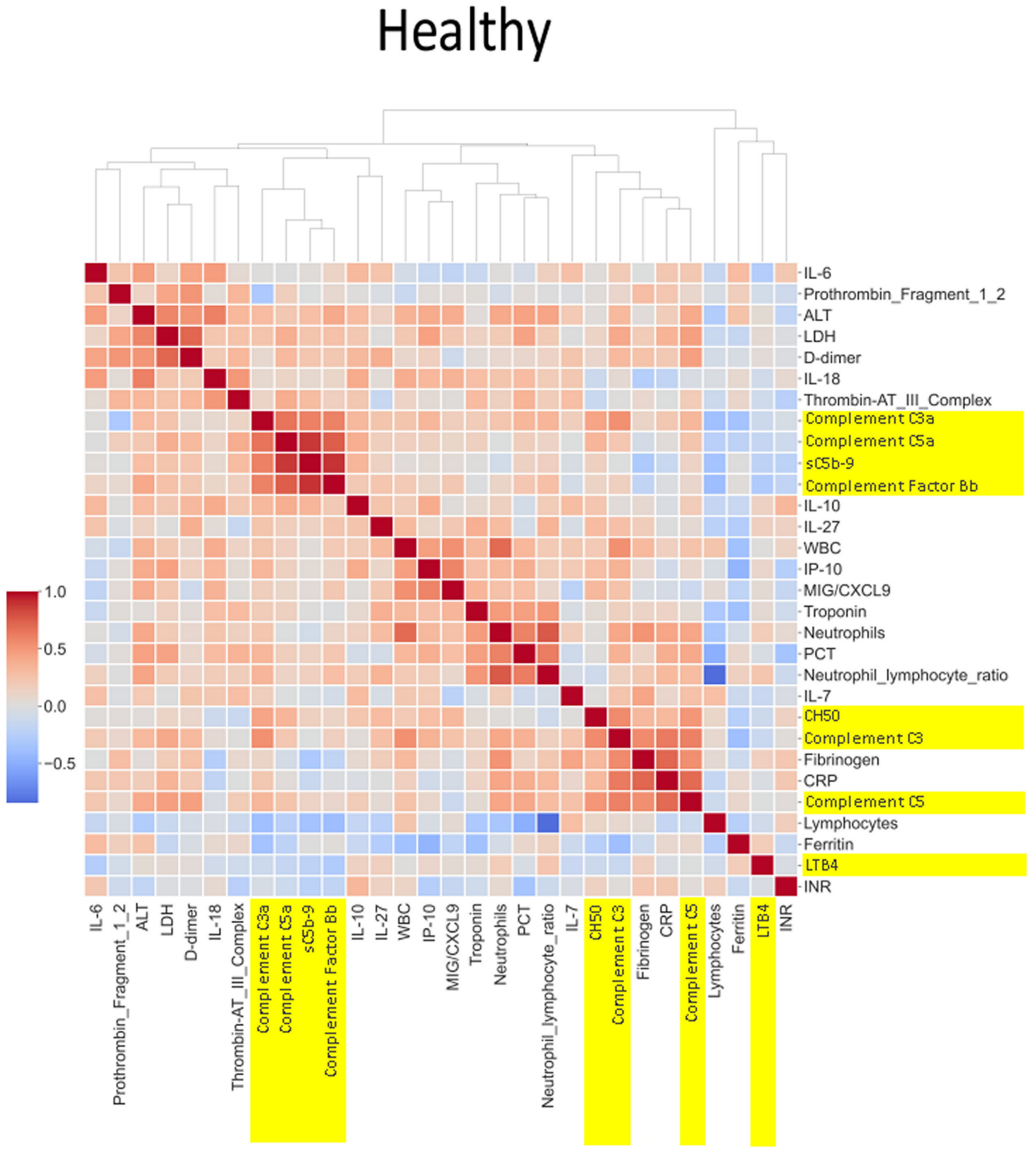
*Correlation and cluster analysis of biomarkers in the CASCADE study grouped by “healthy” classification.* Markers of complement activation are highlighted in yellow. Alanine transaminase (ALT), lactate dehydrogenase (LDH), and D-dimer (remnant protein following fibrinolysis of a blood clot) form one cluster, representing markers of tissue damage. C3a, C5a, sC5b-9, and factor B form another cluster of strongly positively correlated markers. White blood cell count (WBC), interferon-gamma inducible protein 10 (IP-10; a cytokine related to thrombosis), and MiG/CXCL9 (a member of the CXC subfamily of chemokines important in the recruitment of activated T cells to sites of infection) form another cluster where correlation levels are related to each other. CH50 (an indicator of total complement cascade), C3, fibrinogen (coagulation cascade), C-reactive protein (CRP, marker of inflammation), and C5 form another correlation cluster. These clusters (in red hues) indicate biomarkers that increase in their serum concentration together, having an overall additive effect.

**Figure 6:**
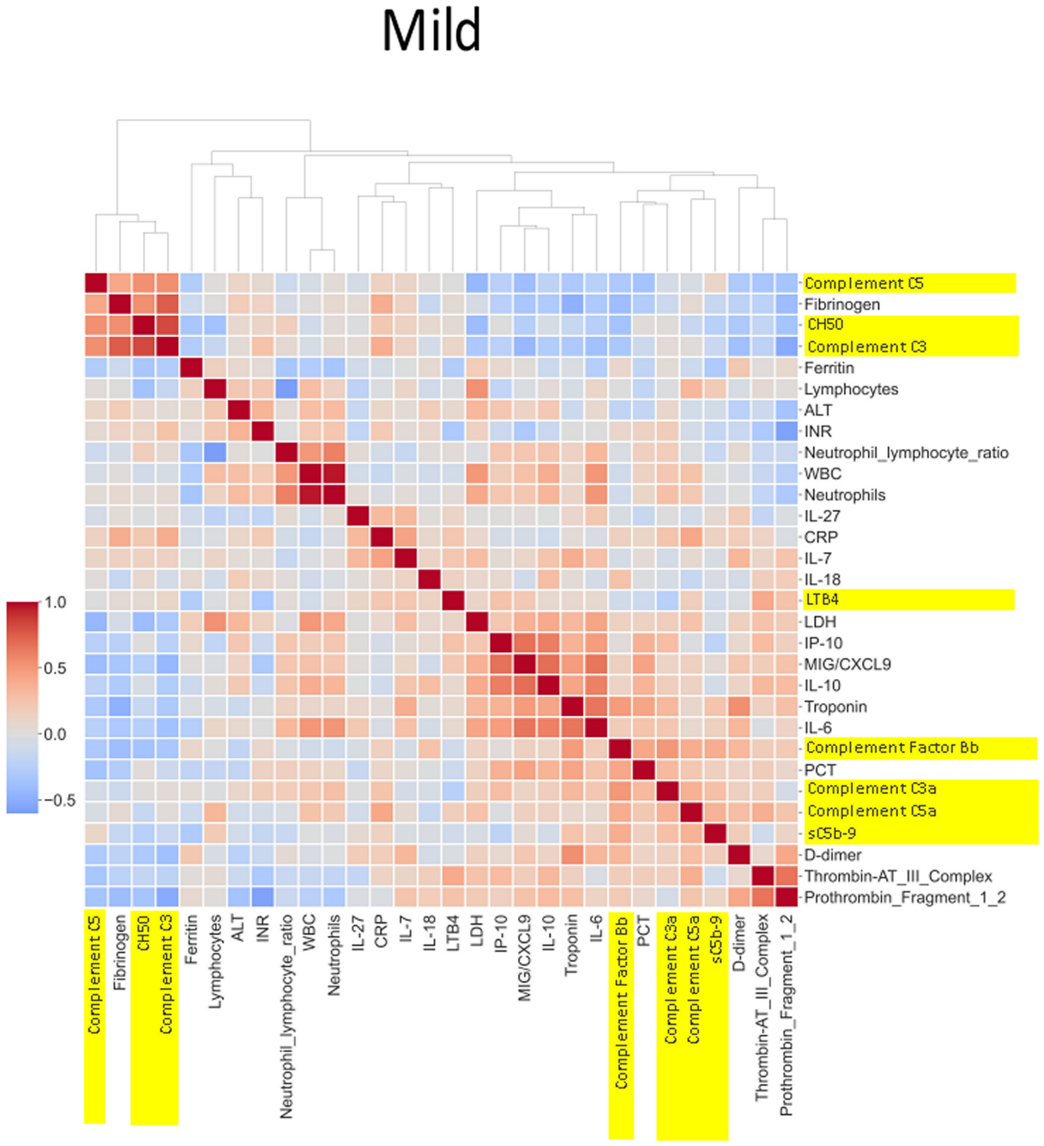
Correlation and cluster analysis of biomarkers in the CASCADE study grouped by “mild” phenotype. ALT is negatively correlated when compared to LDH and D-dimers in the “mild phenotype”. C3a and C5a are also negatively correlated in the mild phenotype; this is in contrast to the trend observed in the “healthy” phenotype. Factor Bb and MiG/CXCL9 appear to share a similar upward correlation in the mild phenotype.

**Figure 7:**
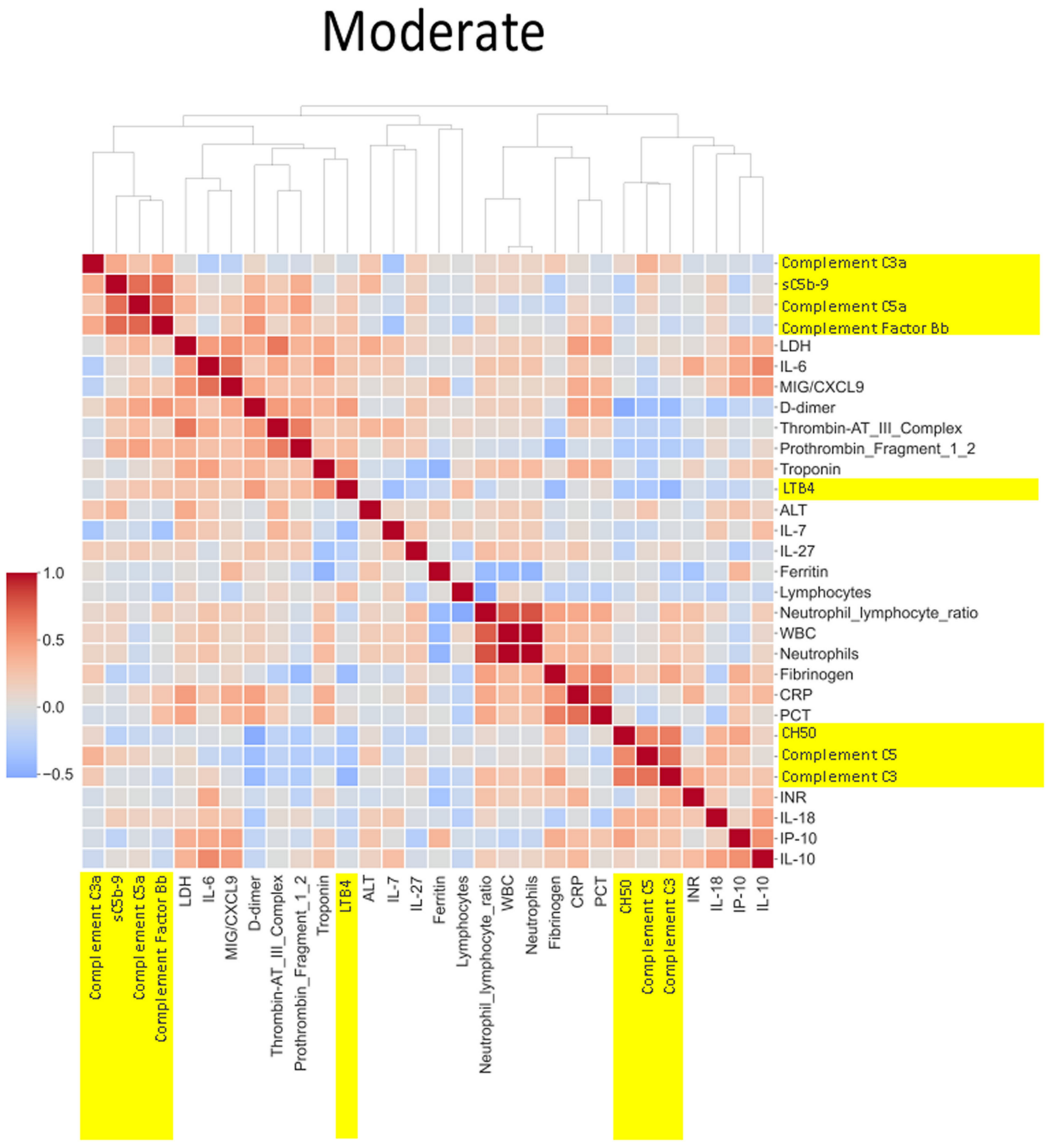
Correlation and cluster analysis of biomarkers in the CASCADE study grouped by “moderate” phenotype. The relationship between C-reactive protein (CRP), a protein produced by the liver in response to inflammation, and the terminal complement complex, sC5b-9, changes from pale red in “mild”, pale blue in “moderate”, and dark blue in the “severe” category. The interpretation from this is that while increases in CRP may follow similar concomitant increases in sC5b-9 in the mild category, increases in CRP may not be reflected by a similar linear increase in sC5b-9 in the severe category. This may account for the consumption of components of the complement cascade in those with the severe category.

**Figure 8:**
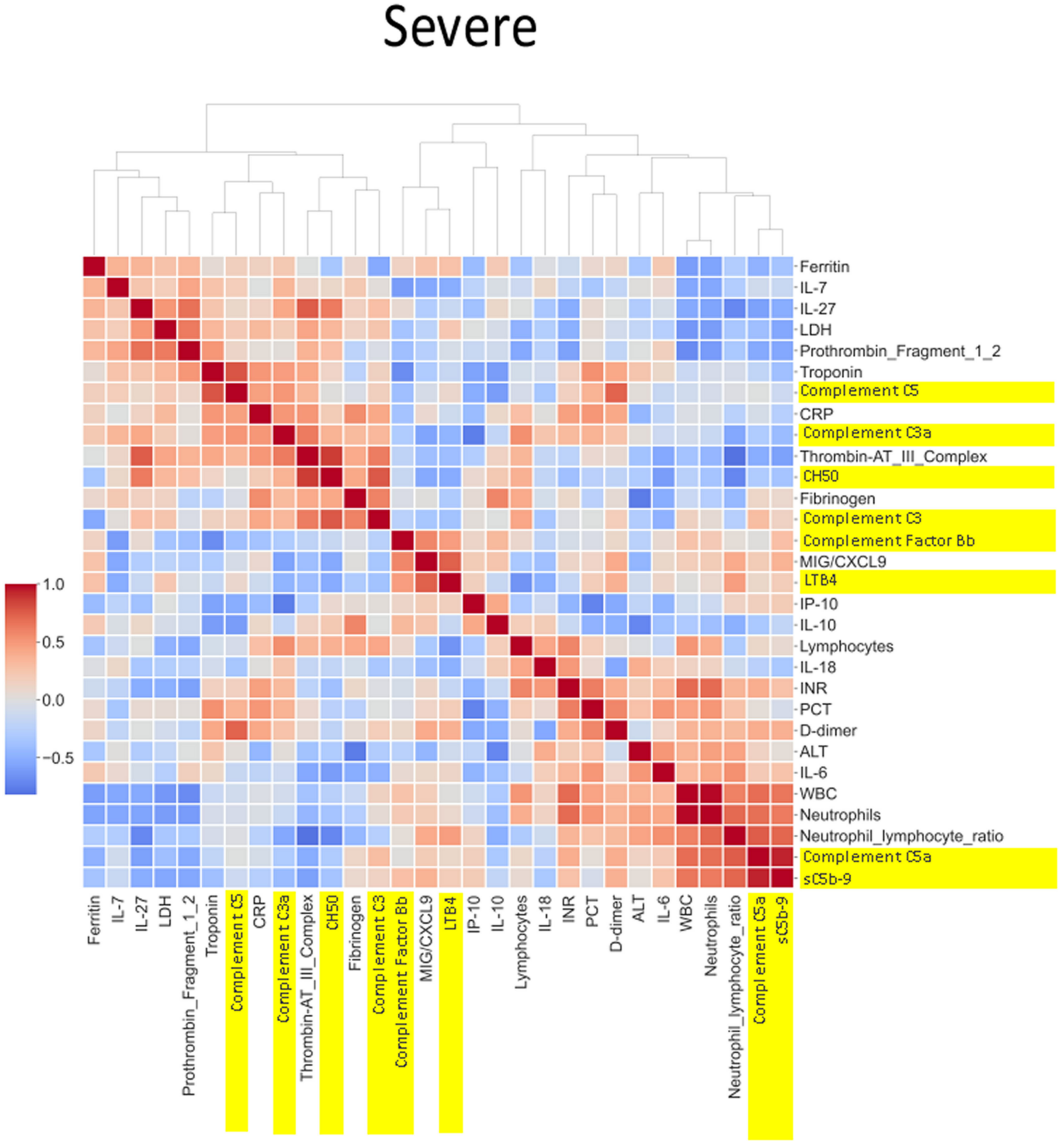
Correlation and cluster analysis of biomarkers in the CASCADE study grouped by “severe” phenotype. sC5b-9 was strongly positively correlated with levels of C5a (a proteolytic fragment from cleavage of C5 by the protease C5 convertase), in the severe category, while similar relationships were seen in the mild category. The level of correlation was weaker (pale red) in the “mild” category. The correlation of C5 to the various biomarkers is grouped by biological function and displayed by the clinical categories of severity. CH50 (a test measuring the activity of all major complement proteins), for example, shows a strong positive correlation in the healthy, mild, and moderate categories but shows a drop in correlation levels in the severe category. One possible reason may be the consumption of complement components by participants within the severe category.

The heatmaps in **Figures 5**–**8** demonstrate a correlation between pairs of biomarkers, with red pixels indicating a positive correlation and blue pixels indicating a negative correlation. Together, the biomarker pairs show an additive effect when they highlight a positive correlation. An additional clustering step, applied to the heatmap analysis, redistributes the biomarker pairs into clusters, where those positively correlated are clustered together while the negatively correlated are grouped together. Cluster heatmaps have an advantage over unordered heatmaps, as they reorder the matrix based on the hierarchical clustering step, thus they display and condense large amounts of rank-ordered data into a compact space. The hierarchical structure is displayed as a dendrogram on the top edge of **Figures 5****–** **8**. This analysis demonstrated that the patient bio-profiles and their biomarkers changed with the severity of respiratory failure. For example, the number of blue pixels increased from **Figures 5** to **8**, and **Figure 8** contained the greatest number of negatively correlated biomarkers (blue).

Neutrophil to lymphocyte ratio (NLR), a known severity predictor in COVID-19 (20), is positively correlated with Complement C5a and sC5b-9 and Complement C5 appears also to be positively correlated with levels of D-dimer (**Figure 8**). NLR was positively correlated with CRP levels in the moderate group (**Figure 7**) while the relationship between NLR and CRP is inversely correlated in the “severe” class (**Figure 8**). Interestingly, complement C5 levels are correlated with levels of troponin in the severe class (**Figure 8**).

IL-27 levels appear to have fewer blue pixels in the mild (**Figure 6****)** (7 blue pixels out of 30 horizontal pixel pairs) and moderate (**Figure 7****)** (6 blue pixels out of 30 horizontal pixel pairs), compared to 12 blue pixels out of 30 in the severe category (**Figure 8****)**. The negative correlation is observed since IL-27 levels appear to decrease with increasing severity; thus, in the pairwise relationship visualised by heatmap analysis, this is displayed as an increase in blue pixels between mild (**Figure 6**) and moderate (**Figure 7**) compared to the severe class (**Figure 8**).

### Linear discriminant analysis and discrimination between non-deteriorators (CASCADE A), deteriorators (CASCADE B), and healthy volunteers (CASCADE H)

**Figure 9:**
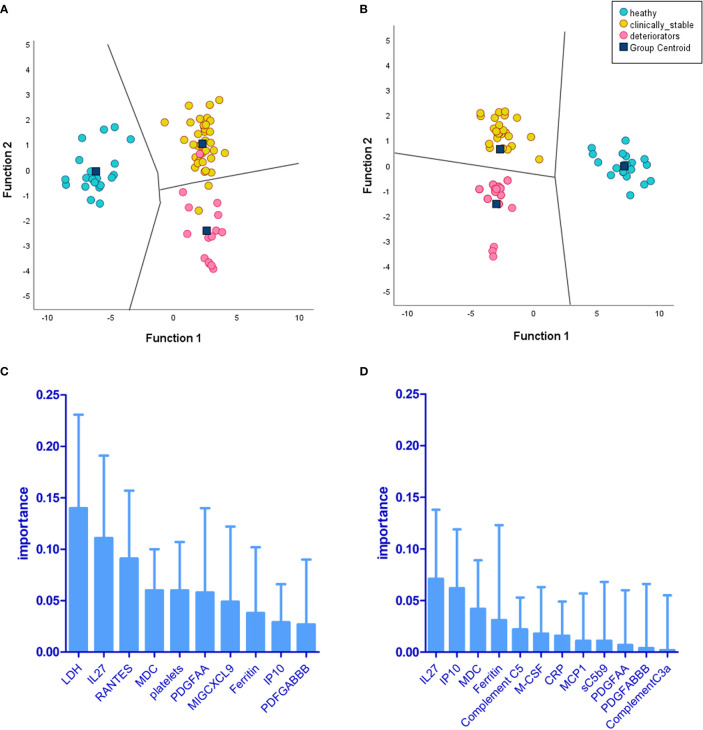
Linear discriminant analysis (LDA) demonstrates levels of biomarkers and cell counts, providing separation of deteriorating patients (CASCADE B) from nondeteriorating (CASCADE A) and healthy participants (CASCADE H). Two LDA models were constructed: **(A)** the territorial map with canonical discriminant functions incorporating timepoint 1 (at admission) of markers for patients in CASCADE A, CASCADE B, and CASCADE H and **(B)** the territorial map with canonical discriminant functions at timepoint 2 of markers for patients in CASCADE A, CASCADE B, and CASCADE H (bloods assayed for markers on day 1 of clinical deterioration of CASCADE B patients). The territorial maps **(A, B)** show separation between the groups based on the ranking of biomarkers **(C)** at timepoint 1 and **(D)** at timepoint 2. There was an increased separation of deteriorating patients (CASCADE B) from clinically stable patients (CASCADE A) and healthy controls (CASCADE H) in LDA model 2 **(B)** without overlap of points between groups in the territorial map areas.

Two separate LDA models are summarised in **Figure 9**. One reflects the immunological landscape of COVID-19 patient predeterioration, and the other is captured from biomarker levels during deterioration. The biomarkers of importance vary between timepoint 1 and timepoint 2; however, some biomarkers appear in both timepoints, although their ranking in order of importance varies between the two (IL-27, macrophage-derived chemokine (MDC), PDGFAA, ferritin, and IP-10). The differences in the order and type of biomarkers provide a discriminative potential between the status of patients at these two time points. IL-27 emerged as the top predictor of clinical deterioration (**Figure 9D**). Complement C5 was among the top five biomarkers of predictive importance (for clinical deterioration) to the model (**Figure 9D**). The metrics for both the LDA models showed 73% accuracy, 77% specificity, and 90.9% negative-predictive value (NPV).

### Biomarker thresholds derived from the LDA models

**Table 3** shows critical threshold values achieving the maximum sensitivity and specificity (associated with the Youden’s index) in both the LDA models, differentiating between the clinically deteriorating subcohort from the nondeteriorating subcohort. The Youden Index is the greatest potential effectivity of a biomarker, a common measure of the ROC curve (21, 22). The threshold values identify a point when the true-positive rate (TPR) is high and the false-positive rate (FPR) is low.

**Table 3:** Threshold levels of biomarkers pertinent in the LDA models.

Biomarker	Threshold value
Deteriorator timepoint 1	
LDH	460 (Units/L)
IL-27	551.15 (pg/ml)
RANTES	338.13 (pg/ml)
MDC	235.44 (pg/ml)
Platelets	200 (×10^9^/L)
PDGFAA	62.92 (pg/ml)
MIGCXCL9	4,453.95 (pg/ml)
Ferritin	80 (mg/L)
IP-10	781.86 (pg/ml)
PDFGABBB	6,154.8 (pg/ml)
Deteriorator timepoint 2	
IL-27	547.27 (pg/ml)
IP-10	599.45 (pg/ml)
MDC	201.52 (pg/ml)
Ferritin	80 (mg/L)
Complement C5	247 (mg/L)
MCSF	7.55 (pg/ml)
CRP	9 (mg/L)
MCP1	24.21 (pg/ml)
sC5b9	1,434.18 (ng/ml)
PDGFAA	72.06 (pg/ml)
PDGFABBB	3,867.24 (pg/ml)
Complement C3a	238.85 (ng/ml)

The level of circulating terminal complement complex sC5b-9 was between 8 and 11 times higher than that reported in normal, healthy donors (23). Threshold values help discriminate between the deteriorating class of patients (CASCADE B) from those who were clinically stable (CASCADE A).

### Comparison of clinical screening tools

The performance of LDA-model 2 against currently available surrogates of clinical deterioration (ROX index, mSOFA score, and NEWS2 score) was compared using ROC-AUC analysis (**Table 4**) using the “training data” of CASCADE A, CASCADE B, and CASCADE H. The performance of the LDA model is represented on the hold-out set as shown in **Table 4** (ROC-AUC analysis using the holdout or test data (20%)).

**Table 4:** Comparison of clinical screening tools with the LDA model.

Model or screening tool	AUC ± SE	95% confidence interval
ROC-AUC analysis on the training data (80%)		
LDA model	1.000 ± 0.000	1.000–1.000
ROX index	0.433 ± 0.106	0.225–0.641
mSOFA score	0.667 ± 0.101	0.469–0.865
NEWS2 score	0.633 ± 0.103	0.431–0.836
ROC-AUC analysis using the holdout or test data (20%)		
LDA model	0.940 ± 0.051	0.862–1.000
ROX index	0.192 ± 0.109	0.000–0.406
mSOFA score	0.827 ± 0.107	0.617–1.000
NEWS2 score	0.654 ± 0.176	0.309–0.999

The mSOFA scores performed slightly better than NEWS2 scores, while LDA model 2 gave an overall superior performance (**Table 4**).

### Machine-learning model for classification of clinical severity by biomarker levels

As explained in **Figure 1C**, the XGBoost model was trained on the CASCADE immune-biological data, to help triage patients by their clinical severity. The model was validated on the hold-out set of data (20%) and gave a prediction accuracy of 83.33%, a positive predictive value of 1.0, a negative predictive value of 0.8, and a Matthew’s correlation coefficient of 0.632.

The XGBoost model was retrospectively used to determine the admission severity of patients in the CORONET study (**Figure 1C**). All patients receiving nomacopan in the CORONET study were classified as “severe” by the model trained on the CASCADE classification.

**Figure 10:**
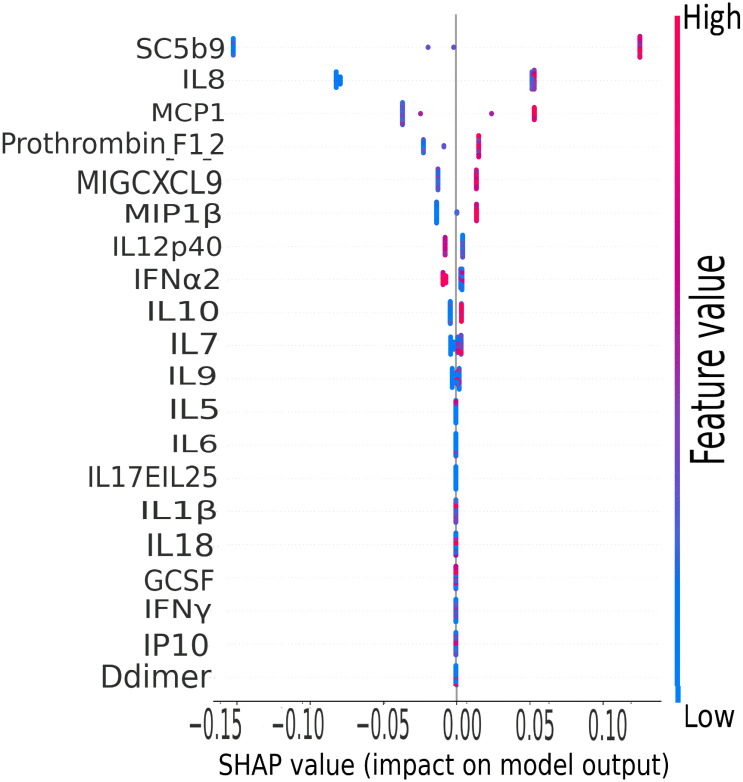
CASCADE machine-learning model on clinical severity by biomarker levels. The ranking of biomarkers in terms of their importance in class prediction is shown as a summary plot of SHapley Additive exPlanation (SHAP) values. SHAP, displays the impact of each feature on the final model. Positive SHAP values mean that a variable has a positive impact on the prediction (in risk prediction, this means that the risk is predicted or present), and negative values imply the converse, meaning a negative impact on the model. The colours represent the concentration value of the biomarkers from low (blue) to high (red). The biomarkers are ordered on the y-axis according to their importance in the predictive property of the model.

The terminal complement component, sC5b9, was the leading biomarker of importance in the classification of clinical severity by biomarker levels in the CASCADE study, as shown by the plot of mean SHAP values (**Figure 10**).

IL-8, a well-studied marker with associations with ARDS and lung inflammation, ranked second highest in its predictive properties and impact on the performance of the XGBoost model (**Figure 10**).

### Nomacopan treatment in the *CORONET study

All nomacopan patients had ROX indices (SpO_2_ <93%) below normal healthy individuals from CASCADE H (**Figure 2**). All were admitted to the ICU, and two patients required early invasive mechanical ventilation. Six out of seven patients survived and were discharged 3 to 22 days after admission with normal CH50 measurements (**Table 5**). One female patient who died had delayed treatment with nomacopan by 3 days.

**Table 5:** Admission and treatment details of patients in the CORONET study treated with the anti-C5 inhibitor, nomacopan.

Patient ID.	Age bin	Sex	qPCR (+ve/−ve)	Admission (month/year)	O_2_ flow rate/min for SpO_2_ >93% [mode of delivery]	Outcome (alive/dead)	Number of days to recovery	CH_50_ at day 3 (U/ml)	Highest C5 (U/ml)	Highest CRP (mg/ml)	Status at discharge
								UL = 40 U/ml	UL = 10 mg/ml	UL =10 mg/ml	
1	5	Male	+ve	September/2020	8L [NC]	Alive	11	<17.0	39.0	40.5	Normoxia on air
2	4	Male	+ve	November/2020	2L [NC]	Alive	3	22.0	40.0	199.0	Normoxia on air
3	3	Male	+ve	January/2021	6L [NC]	Alive	5	21.0	26.0	116.0	Normoxia on air
4	3	Male	+ve	February/2021	6L [IMV 4 days, then NC]	Alive	22	20.0	54.0	193.0	4L O_2_ at rest, 8–10 L on exertion
5	3	Male	+ve	April/2021	4L [NC]	Alive	5	15.0	36.0	109.0	Normoxia on air
6	5	Male	+ve	July/2020	4L [NC]	Alive	6	<10.0	22.2	154.18	Normoxia on air
7	1	Female (delayed Tx nomacopan for 3 days)	+ve	July/2020	[IMV 7 days postadmission]	Dead 14 days postadmission	N/A	>60.0 prior to nomacopan Tx	24.3	274.65	Dead 14 days postadmission

IMV, intubated and mechanically ventilated; UL, upper limit; NC, nasal cannulae; N/A, not applicable; qPCR, quantitative polymerase chain reaction; Tx, treatment. Age bins: 20–29 = 1, 30–39 = 2, 40–49 = 3, 50–59 = 4, 60–69 = 5, 70–79 = 6. The primary endpoint was full respiratory recovery, defined as nondependence on mechanical ventilation and SpO_2_ of >93% by pulse oximetry, breathing air without the need for supplementary oxygen. Secondary endpoints included treatment-related adverse events, time to SpO_2_ >93% with no oxygen supplementation, ventilator-free days between Day 0 and discharge, and duration from hospital admission to discharge and recovery. Treatment lasted for a maximum of 14 days.

## Discussion

The CASCADE study carried out in the UK was designed to determine if the separate classes (“healthy”, mild, moderate, and severe) of COVID-19 patients determined by clinical judgement and risk assessment would reveal distinct “immunological fingerprints”, by biomarker levels. Our findings have shown such fingerprints exist, and when the classification is automated *via* machine-learning algorithms, it would serve as a rapid risk assessment and triage tool. Secondly, distinct immunological fingerprints in COVID-19 patients were determined, allowing early identification of patients who might have the propensity to deteriorate clinically.

The CORONET study carried out in the USA applied a similar but smaller panel of biomarker assessment, but crucially, this study recruited seven patients with severe symptoms who were treated with an off-label complement C5 inhibitor, nomacopan, administered under specially procured permissions under the “compassionate-use” regulations from the FDA.

The two studies intended to determine the “immunological fingerprint” of patients categorised by their symptoms, unique to the clinical severity demonstrated during their hospital admission for COVID-19. Clinical-triage efforts during a pandemic can be a huge responsibility and burden on senior clinical physicians; however, if the risk assessment were based on bioprofiling using serum biomarker levels and aided by an artificial-intelligence-based model, this could be implemented by a wider, less specialist workforce.

The correlation coefficients displayed in the heatmaps describe the direction of the relationship between pairs of variables, where a positive correlation means that the pairs of variables are either both high or both low at the time of biomarker measurement. This is represented as red hues in the heatmap analysis. A negative correlation occurs when one variable is high and the other is low at the time of biomarker measurement; these are represented as blue hues in the heatmap analysis.

The heatmap analysis displays a “bird’s eye view” of an immunological fingerprint by comparing pairs of variables within each severity classification. Clusters with red pixels indicate variable pairs that are increasing or decreasing in their values together. The blue pixels indicate values in opposite directions to each other. The paired relationships shown here are primarily a statistical representation of the direction of the relationship in the biomarker levels; they may not necessarily reflect a biological relationship where the level of one biomarker affects the direction of the other.

An example of where the levels of one biomarker might impinge on the levels of another is illustrated in the relationship of the neutrophil-lymphocyte ratio (NLR) to CRP levels. Our analysis shows that NLR is positively correlated to CRP levels in the moderate group (**Figure 7**). The importance of both these biomarkers has been shown in a separate study, where the utility of these markers are excellent diagnostic predictors of COVID-19 in a binary logistic regression model (24). However, our heatmap analysis adds a very important angle to the immunological biomarker study since **Figure 8** shows the NLR ratio is inversely correlated to CRP in the “severe” class. This conflicting observation (between that in the moderate vs. severe) could be explained as follows: neutrophil concentrations when high, (as in the “severe” class), restrict viral replication *via* degranulation, phagocytosis, and the release of neutrophil traps and subsequently reduce viral titres with a concomitant lowering of CRP values as have been reported previously (25). Thus, the “compartmentalisation” of the immunological fingerprints in COVID-19 through the mild, moderate, and severe severity classes are captured uniquely in our use of heatmaps as a visual representation. The association of specific levels or ranges of cytokines with disease severity is not a new concept and has been described before in the literature (26, 27).

The release of neutrophil elastase from highly activated neutrophils mediates lung injury in sepsis, including COVID-19 (28), and inhibitors to neutrophil elastase (sivelestat) are protective in inflammatory lung injury (29), and have been proposed to be beneficial in COVID-19-related lung injury (30). Increased levels of circulating neutrophil elastase have been shown to correlate with complement activation (31). Both neutrophils and C5a (a surrogate marker of complement activation) are raised in the severe phenotype in the CASCADE study (**Figures 3**, **4**), and they show a positive correlation in the heatmaps (**Figure 8**); therefore, the severity of inflammation in COVID-19 patients due to complement activation might be attenuated by the release of neutrophil elastase in COVID-19.

Significant elevations of cardiac troponin-T, correlating with an increased risk of cardiac damage, have been reported in COVID-19 (32, 33). In mouse models of myocardial ischaemia/reperfusion injury, in the absence of viral infection, C5 levels were elevated along with increases in serum troponin levels (34). Our heatmap analysis shows elevated serum complement C5 is positively correlated with raised troponin levels. This finding could be explained by observations from other studies where the high inflammatory burden from the spectrum of cytokines and complement proteins released in the disease is thought to induce myocardial injury (35). Thus, the heatmap analysis provides a unique “aerial perspective” of the “immunological landscape”, within the boundaries of clinical severity types, to help guide the clinician and investigative scientists, of the potential trend various markers are adopting during hospitalisation and treatment of a patient with COVID-19.

IL-33, a member of the IL-1 cytokine family, has been suggested to play an important role in severe COVID-19 (36), upregulating Th-2 cytokines such as IL-5 and IL-13 (37–39). The cytokine panel used in our study did not contain assays for IL-33; however, as IL-5 and IL-13 were surrogates of IL-33 activation, there was a significant increase in IL-5 levels above normal levels, although no concomitant increase of IL-13 was observed.

LDA model 2 (**Figure 9D**) identified IL-27 and IP-10 as the top two predictors of clinical deterioration in COVID-19. A decrease in the levels of IL-27 has been shown in other studies as a reliable predictor of adverse clinical outcomes in COVID-19 (40). Our heatmap analysis captures the trend of IL-27, with fewer negatively correlated pairs with IL-27 in the mild and moderate categories compared to the severe category (12_blue pixels_/30_total horizontal pixels_). IP-10 has been demonstrated in a previous study as a marker associated with clinical severity in COVID-19 and correlated with disease progression (41), and it is interesting that our model identified this as a key marker in the recognition of a patient with the propensity to deteriorate clinically.

MDC was identified as third in order of importance in our LDA model (**Figure 9D**). MDC has been shown to inhibit the replication of CCR5-dependent HIV in macrophages (42) and play a protective role over CD4^+^ T cells from infection by HIV (43). Our findings show that the levels of MDC are significantly lower than that found in normal, healthy individuals (**Figure 3**), and this is in agreement with a previous study that reported reduced levels of the chemokine in COVID-19 patients (44).

Raised ferritin levels have been observed in a range of inflammatory diseases (45), and intracellular ferritin is thought to leak into serum from damaged intracellular stores (46). Ferritin was identified as fourth, in order of importance, in our LDA model in timepoint 2 (**Figure 9D**). The presence of excess iron in the internal milieu is known to favour the growth of numerous viruses (47); this might explain why the order of importance of ferritin in timepoint 2 (point of clinical deterioration) is moved up by rank compared to that in timepoint 1. The role of ferritin as an important biomarker in the progression of the disease in COVID-19 has been reviewed previously (48).

COVID-19 patient levels for CH50, sC5b-9, C5a, C5, C3a, C3, and factor B were all higher than levels in healthy individuals (**Figure 4**). Complement C5 and CH50 levels correlate with CRP levels in all severity classes, as shown in our heatmap analysis (**Figures 6****–** **8**). Complement activation has been documented to positively correlate with CRP levels since CRP activates complement *via* the classical pathway, activating C1q, which then activates the rest of the complement cascade (49).

The XGBoost model highlighted that IL-8 ranked second highest as a predictive variable (**Figure 10**). IL-8 is a well-studied neutrophil chemotactic factor that plays a key role in numerous pathological conditions. IL-8 is expressed in neutrophils, epithelial cells, hepatocytes, fibroblasts, endothelial cells, and alveolar macrophages (50, 51). It has been proposed that the presence of IL-8 in the bronchoalveolar lavage fluid (BALF) is a useful prognostic variable in ARDS patients (50). Furthermore, IL-8 has been implicated in the recruitment of neutrophils to the lungs in acute inflammation of the lung (52). IL-8 has been associated with the development of respiratory failure following the reduction of PaO_2_/FiO_2_ (53), and the reduction in lung oxygenation levels across the severity classes has been demonstrated in this study by our analysis of ROX scores (**Figure 2**).

MCP-1 was ranked third in the XGBoost model (**Figure 10**); this is also in agreement with the findings of other groups where MCP-1 expression levels were found to be higher in patients with COVID-19, especially those admitted to intensive care units (54). Increased levels of MCP-1 have been isolated in lung tissue of COVID-19 patients (55). It has been suggested from other studies that monitoring MCP-1 levels during hospitalisation could help prevent COVID-19 from progressing from a mild to a severe presentation (56); this finding is coherent with our XGB model of stratification of risk classes and the prominent ranking of this marker in the predictive order of importance.

The CORONET study was a “compassionate use” study, allowing off-label nomacopan treatment; nomacopan is currently in phase III studies for a coagulopathic disease, thrombotic microangiopathy, in children treated with human stem cell transplants (57). COVID-19 coagulopathy has been well studied (58), and a marker associated with coagulation (prothrombin F12) has been identified as key in our XGBoost risk stratification model (**Figure 10**).

Six patients received nomacopan on admission, and one patient’s treatment was delayed (for nonmedical reasons) for 3 days and died from COVID-19-related complications. The remaining six CORONET patients at admission presented with ROX scores consistent with severe hypoxaemic respiratory failure (**Figure 2**). All surviving patients were discharged home between 3 and 22 days after admission with high circulating levels of sC5b-9 but normal CH50 values.

The terminal complement complex exists in two forms: soluble sC5b-9 and the MAC. C3 convertase cleave C5 to C5a and C5b. C5b initiates the activation of the MAC that includes C6, C7, C8, and C9 complement components important for allowing the MAC complex to penetrate the cell membrane (59). The bound membrane attack complex forms a cytolytic pore. The complement regulators, CD46, CD55, and CD59, are important for controlling complement activation and prevention of the MAC pore assembly in the cell membrane (60).

Although pre-nomacopan treatment levels of CH50 of patients in the CORONET study were not available for comparison to the day-3 levels of CH50, the pre-nomacopan treatment C5 levels (all above upper limits) hint that complement components were high in the systemic circulation at admission (**Table 3**). CH50, in the CORONET study, is a useful index for identifying people at risk of deteriorating respiratory failure in COVID-19 pneumonia. Hospital-discharged nomacopan-treated CORONET patients appear to be healthy and stable without any long-term or residual sequelae. While these are preliminary results on a small-sized “compassionate-use” cohort, it warrants a randomised controlled trial of nomacopan for minimising the progression of severe lung injury in COVID-19.

One drawback of clinical risk stratification in a “resource-poor” and extremely busy pandemic setting is the dependence on senior clinical reviews, whereas automated algorithms trained on serum biomarker evaluation, could be implemented by a larger portion of the nursing workforce (61), particularly in precision nursing, with less pressures on senior clinical staff (61, 62).

Thus, the LDA model, based on biomarker profiling of COVID-19 patients, could identify patients with the propensity of deteriorating clinically. The dimensionality-reduction feature of LDA allowed a minimal set of biomarkers to be used as a prognostic guide; this is both advantageous clinically (less laboratory resources) and economically, as fewer markers mean lower costs. The XGBoost model is also useful to triage patients according to the mild, moderate, and severe categories based on biomarker profiles; such models could be easily implemented by nonspecialist staff during a pandemic.

These models were generated based on biomarker profiles from patients of the first and second COVID-19 waves, and while these bioprofiles look at the downstream translational or proteomic signature, we do not know if the proteomic signature might be affected by future variants of the SARS-CoV-2 virus.

Nevertheless, there is a need to improve the management of resources during a pandemic, and several studies have proposed exploring the use of digital health solutions and artificial intelligence in management and triage situations (63). Lung diseases carry significant mortality and morbidity worldwide, and COVID-19 is a sentinel example of the need to expand the utility of biomarker diagnosis in diagnosis and triage situations (64).

## Limitations of the study

The biological lability of complement components and leukotriene B4 in blood were confounding issues for assessing patient outcomes. The average age of participants in CASCADE A and CASCADE B was in their sixth decade, and it was very difficult to obtain age-matched healthy controls in their sixth decade without significant comorbidities, particularly during a pandemic period where a large proportion of the elderly were self-isolating. We recognise the effect of age and gender on the components of the immune system and acknowledge this limitation. Our studies are underrepresented by minority ethnic groups due to the population demographics of our local hospitals. Future studies in the validation of the models and trials of nomacopan should address these issues. Future studies should be undertaken with significantly larger sample sizes.

## Data availability statement

Requests for raw data pertaining to the study can be communicated to the corresponding author, they will be reviewed following agreements.

## Ethics statement

The studies involving human participants were reviewed and approved by South-Central Berkshire Research Ethics Committee (REC reference: 20/SC/0228 May 22nd, 2020) and Investigational New Drug’ (IND) application approved as an “Expanded Access Application” by the FDA. The patients/participants provided their written informed consent to participate in this study.

## Author contributions

AC, LW, TB, LGD, TH, JB, CC, JM, DG, CR, WW-D: conceived the study and delineated the hypothesis and designed the study. AC, LW, TB, JB, CC, JM: made final decisions regarding case selection and inclusions. LW, LGD, TB, DG, JB, CC, JM: collected samples from patients and facilitated downstream biological processing. LGD: implemented the machine-learning and neural net aspects of the study and conducted statistical analysis of the study. LGD is a fellow of the Royal Society of Statistics. All authors read and edited the drafted manuscript for intellectual content and discussed the data interpretation. All authors approved the final version of the manuscript.
