# Supplementary material for: Clinical severity classes in COVID-19 pneumonia have distinct immunological profiles, facilitating risk stratification by machine learning
Source: Front Immunol. 2023 Sep 5;14:1192765. doi: 10.3389/fimmu.2023.1192765 (PMC10508987; doi:10.3389/fimmu.2023.1192765)
Supplement: Supplementary file 1 [file DataSheet_1.pdf]

ONLINE DATA SUPPLEMENT

Inclusion-exclusion criteria.

Supplementary Table 1. Inclusion and exclusion criteria for CASCADE and CORONET

Inclusion criteria:
i) Adults ≥18 years old requiring hospital admission for COVID-19<br>ii) COVID-19 confirmed either by a positive swab (using RT-PCR) or based on a high level of clinical probability confirmed by the presence of typical symptoms and compatible radiological findings on imaging with no alternative cause for these findings identified by the treating physician
Exclusion criteria:
1. Renal replacement therapy on ITU<br>2. Significant trauma (including an acute fracture or significant head injury)<br>3. Massive transfusion of blood products<br>4. Confirmed bacteraemia with pathogenic organism on blood cultures or other severe bacterial infections (including abscess/empyema) which persist despite broad-spectrum antibiotics and are thought to be significantly contributing to the patient's symptoms and clinical state. Recruitment will not be delayed however pending a negative culture).
Healthy volunteers:
1. Adults ≥18 years old with a negative RT-PCR swab<br>2. no new cough, fever, or flu-like symptoms within the preceding 4 weeks<br>3. no significant comorbid conditions or requirement for regular medications.<br>4. able to provide informed consent

Bioprocessing of clinical samples

Combined nose and throat swabs for COVID-19 RT-PCR were obtained from patients presenting to hospital as part of routine clinical practice. Swabs were obtained from healthy volunteers (pre-screened for COVID-19 RT-PCR with a confirmed negative result). Swabs samples were transferred to the Diagnostic Microbiology Laboratory at Portsmouth Hospitals University NHS Trust in Viral Transport Media (VTM). Samples were analysed on a Hologic- Aptima® SARS-CoV-2 Assay (Panther® System) as per manufacturers guidelines (AW-21491-001 Rev. 002).

Bloods samples were obtained by venesection from participants at recruitment. In the event of deterioration with escalation of severity category, repeat samples were obtained, with up to 2 additional sampling points but not more than one sample per day. Routine biochemistry and haematology samples were processed at Queen Alexandra Hospital in line with standard laboratory operating procedures. All research samples were treated as infectious samples and processed with the necessary precautions and handled under containment level 2 conditions in line with Public Health England guidance. Samples were left to clot for 30 minutes and then immediately centrifuged (2000xg for 10 minutes). Samples were spun for a further 15 minutes, to ensure clear separation of cellular material from serous fluid. Serum and plasma samples were snap-frozen promptly on the day of

venesection and stored at -80°C. Samples were then shipped on dry ice in line with national guidance to external laboratories for analysis.

Laboratory analysis of biomarkers from blood and swab samples.

Serum and plasma samples were analysed for an extensive panel of cytokine, chemokine and endothelial dysfunction measures, markers of complement activation, extended coagulation measures, and levels of leukotriene B4, alongside routine clinical markers of coagulation and inflammation.

The laboratory personnel carrying out the assays were blinded to the clinical information pertaining to each case. A panel of 48 different inflammatory mediators were measured in serum using the human-cytokine 48-plex discovery assay (Eve Technologies, Canada). Markers of complement activation CH50, C3, C5, C3a, C5a, Bb and the terminal complement complex SC5-b9 were quantified using the Quidel Microvue ELISA kits (The Doctors Laboratory, London).

Selection of algorithms for analysis.

Nine machine-learning algorithms and one Multi-layer Perceptron (deep neural net) algorithm were screened against the training-dataset to choose the algorithm with the best performance in prediction accuracy during the cross-validation steps.   This is illustrated in Supplementary Figure 1.

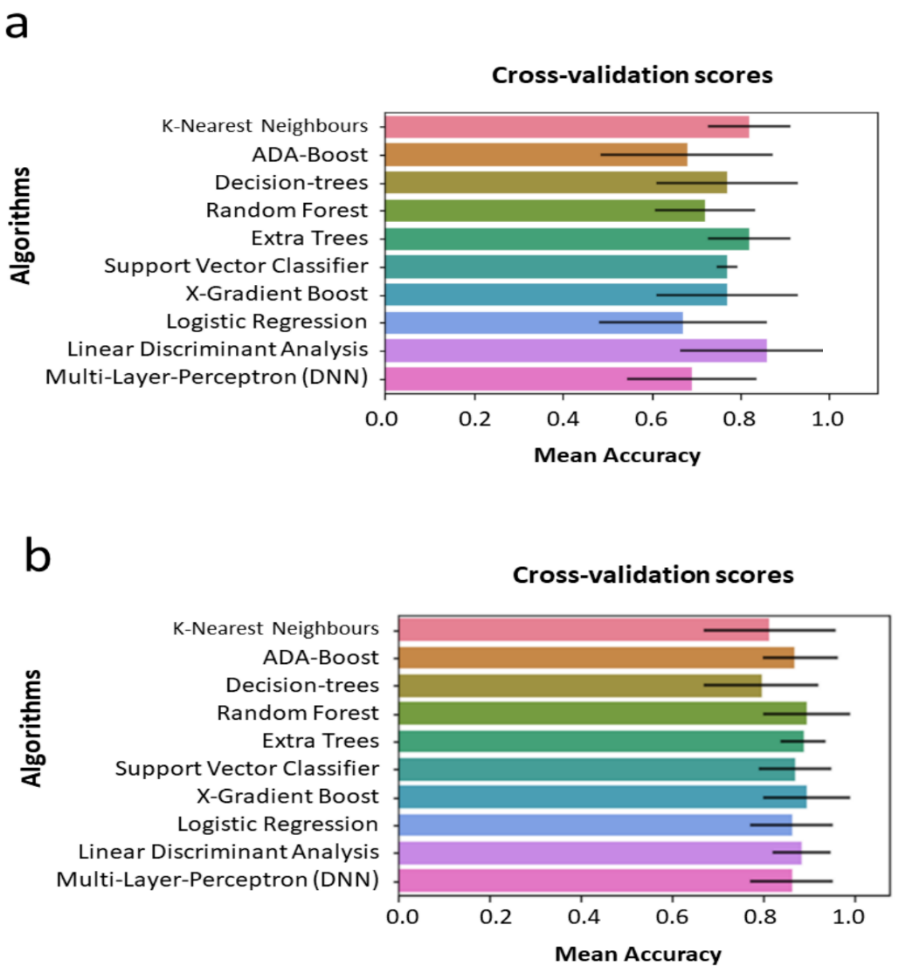

Supplementary Figure 1. Screening of algorithms to select the best model for predictive performance. Nine machine learning algorithms and one multi-layer perceptron (DNN) algorithm were screened against the training-dataset for the predictive performance on the data-slices generated during cross-validation steps. Mean Accuracy is displayed on a scale of 0 to 1.0, where an accuracy of 1.0 indicates perfect prediction accuracy. Error bars are represented by the standard error of the mean. (a) When the CASCADE data was split into deteriorators, non-deteriorators and healthy individuals, the linear-discriminant analysis (LDA) algorithm gave the best metrics in terms of prediction accuracy. (b) When the CASCADE data was classified into "severe" and "non-severe" cases, the XGBoost algorithm yielded the best metrics in terms of prediction accuracy.

Machine-learning: Linear Discriminant Analysis (CASCADE study)

Linear discriminant analysis is an established machine learning algorithm and a powerful method for dimensionality reduction, where data is transformed from high to a low-dimensional space, the transformation retains salient information of the original data. In simple terms, what this means is that the method reduces the number of variables from the model, in the CASCADE study, this means that the method retains the most important variables which confer predictive properties to the model while less important variables are removed. The method is also useful in removing "noise" from the data and helps diminish overfitting.

Two Linear discriminant analysis (LDA) models were generated, coded in Python 3.8, using Scikit-learn (ver.0.16.1). An 80:20 train-test split was implemented for model 1 (timepoint 1) and model 2 (timepoint 2), this ratio was decided on extensive evaluation of the accuracy and other metrics of the model during the cross-validation stages. Hyperparameters were optimised and determined following extensive experimentation and testing of models, while employing a grid-search algorithm.

For both model 1 and model 2, shrinkage was set at 0.005 and the optimum solver following hyperparameter optimisation was the least-squares method. Shrinkage is similar to the L1/L2 penalty terms used for regularizing and providing a better fit. The reason for shrinkage and regularisation is to minimise overfitting, in the aim to build a robust and accurate model. Overfitting is a phenomenon observed in machine-learning and statistics when a model fits the training data extremely well, so that it cannot generalize to new, unseen observed data. The parameters for shrinkage were determined following extensive testing (between 0.0000001 and 1 in 0.0000005 incremental steps).

The training data following train-test split comprised of the following membership:

i) Healthy – 16
ii) Clinically stable – 28
iii) Deteriorators – 13

Synthetic Minority Over-sampling Technique (SMOTE) was implemented to address the class imbalance in the training data. The class targeted for resampling was set at "auto", equivalent to "not-majority". The parameter "nearest neighbours" used to generate the synthetic samples was set at 5, this value was determined following extensive experimentation, testing and evaluation of the cross-validation metrics.

Supplementary Table 2. Metrics on hold-out (test) dataset. Metrics shown here are for LDA model 1 (time-point 1, admission) and LDA model 2 (time-point 2, point of deterioration).

	LDA model 1	LDA model 2
accuracy	73.33%	73.33%
Specificity	76.92%	76.90%
False positive rate	23.08%	23.08%
Negative predictive value	90.91%	90.90%

CASCADE model for prediction of risk of clinical deterioration

The final predictive LDA-model2, with just the top-5 variable predictors (IP10, IL27, Ferritin, MDC, CRP and Complement C5) was encrypted, and deployed as a secure web-app, using STREAMLIT for review at:
https://darthcruz3-cascade-co-lgd-ldamodel2-frontiersimm-23mar23-u2c2fv.streamlit.app/

The reduced features to just the top 5 variables is shown here, since a reduced feature/variable set is more likely to be clinically deployable.

Machine-learning: XGBoost model (CASCADE study)

Patients within the "mild" and "moderate" classes were re-classified as group-1 (non-severe), while patients in the "severe" class were re-classified as group-2. Class imbalance was resolved using SMOTE.

Data was split into train: test sets using an 80:20 ratio. Nine machine-learning algorithms and one neural-net algorithm (multi-layer-perceptron) were screened using the k-means stratified cross-validation (number of splits = 5). Mean-accuracy was used to evaluate the performance of the algorithms.

Hyperparameters were tuned using a grid-search method. The optimally tuned hyperparameters for the XGBoost algorithm are shown below in Supplementary Table 3.

Supplementary Table 3. Optimum hyperparameters for the XGBoost algorithm

**Hyperparameters**	**value**
Colsample_bytree	0.7
Learning_rate	5
Max_depth	6
Min_child_weight	11
N_estimators	100
N_thread	4
Objective	Multi-softmax
Subsample	0.8

The ROC-AUC score=0.9, confirmed the superior performance of the XGBoost algorithm on prediction of the hold-out or test dataset.
